# Antifungal activity of recombinant thanatin in comparison with two plant extracts and a chemical mixture to control fungal plant pathogens

**DOI:** 10.1186/s13568-018-0710-4

**Published:** 2018-11-02

**Authors:** Mojtaba Mamarabadi, Abbas Tanhaeian, Younes Ramezany

**Affiliations:** 1 Department of Plant Protection, Faculty of Agriculture, Ferdowsi University of Mashhad, Mashahad, Iran; 2 Department of Biotechnology and Plant Breeding, Faculty of Agriculture, Ferdowsi University of Mashhad, Mashahad, Iran

**Keywords:** Phytopathogenic fungi, Plant diseases control, Antimicrobial peptides, Tomato early blight

## Abstract

The most common method for controlling plant diseases is the application of chemical pesticides and sometimes use of resistant cultivars. Due to the effects of chemical pesticides on human and environmental health, mutation in pathogens and resistance to various toxins besides the challenges with resistant cultivar production, the constant use of these methods are not recommended any longer. Thus, use of biological control agents along with the natural ingredient extracted from plants and application of peptide with antimicrobial activity, have been the focus of many researchers. In the present study, the antifungal activity of two plant extracts named Turmeric and Persian lilac in comparison with a chemical mixture and recombinant thanatin were evaluated against five following fungal plant pathogens; *Geotrichum candidum*, *Botrytis cinerea*, *Rhizoctonia solani*, *Alternaria tenuissima* and *Gibberella fujikuroi*. The results showed that, all treatments have antifungal activity against tested fungi. Both plant extracts were shown an acceptable antifungal activity against tested fungi but their inhibition effects was not comparable with chemical mixture. Turmeric showed a higher rate of mycelial inhibition than Persian lilac. Amongst all treatment, thanatin showed a great antifungal activity by its application at µg level under both in vitro and in vivo condition. Considering to the compatibility of thanatin with human health and environmental safety we could imagine a clear perspective for the application of this recombinant peptide in sustainable agriculture.

**Electronic supplementary material:**

The online version of this article (10.1186/s13568-018-0710-4) contains supplementary material, which is available to authorized users.

## Introduction

The main source of income for the half people from the world’s population comes from agriculture. The damage caused by plant pathogen and pest makes a significant failure in the grower’s income.

The pesticides are used to prevent the reduction of crop production each year. Evaluating the environmental risks associated with pesticide application indicates that these problems have been increasing over time and this is because of the increasing amount of research on the environmental impacts of pesticides in the world (Pingali and Roger 2012). The most common method for controlling plant pests and diseases in sustainable agriculture is the minimal application of chemical pesticides and using resistant cultivars as well (Stenberg 2017). Due to the effects of chemical pesticides on human and environmental health, mutation in pathogens and resistance to various toxins, the continued use of chemical and synthetic pesticides is not a suitable option (Carvalho 2017). Moreover, there are numerous challenges in resistant cultivar production (kossmann 2012). Thus, other solutions such as using biological control agents along with further consideration on the plant natural ingredients were suggested. Worldwide studies have shown that plant extracts have a high ability to inhibit the growth of microorganisms, and in this regard, medicinal plants have been widely used as antimicrobial agents (Hafidh et al. 2011). Turmeric or “Indian saffron” (*Curcuma longa* L.) is a plant native to Southeast Asia and belongs to the family Zingiberaceae (Aggarwal et al. 2007; Avanco et al. 2017). The rhizomes of this plant is rich in curcumin which is a natural pigment with important biological activity (Martinez-Correa et al. 2017). Turmeric has been observed to be toxic against fungal pathogens caused different plant diseases (Ferreira et al. 2013; Avanco et al. 2017). We also used Persian lilac as another plant extract with antimicrobial activity. Persian lilac (*Melia azedarach* L.) is one of the most useful plants used in various parts of traditional medicine and belongs to the family of Meliaceae (Saleem et al. 2008; Neycee et al. 2012). The reason for the use of Persian lilac extract in the present study was the likelihood and potential of these compounds for plant pathogen inhibition. For example, the anti-growth effects of this plant extract have been confirmed and reported on *Aspergillus niger*, *Aspergillus flavus*, *Fusarium oxysporum* and *Rhizopus stolonifer* (Sen and Batra 2012).

Besides the antimicrobial effect of plant extracts for fungal inhibition, other alternative methods like use of antimicrobial peptides (AMPs) have also been considered. Many attention have been paid by the researchers on AMPs in recent years. This is due to their efficiency against different pathogens. These peptides are found in nature and have been isolated from a wide range of organisms. Many plants and animals have been manipulated with AMPs encoding genes and several pesticides have been produced based on these peptides. There are many successful examples for application of these peptides in agriculture which are indicated a promising future for extensive application of AMPs (Zasloff 2002; Montesinos 2007; Keymanesh et al. 2009).

Among different Amps, a recombinant thanatin has been synthetized and evaluated in this study. Thanatin was originated from the spined soldier bug (*Podisus maculiventris*). It is the first inducible insect peptide with a broad range of activity against bacteria and fungi at physiological concentrations. Thanatin contains two amino acids including two cysteine residues that form a disulfide bridge (Mandard et al. 1998). So far, several studies have been performed on this peptide. For example, the transgenic rice encoded thanatin was resistant to blast disease in field evaluations (Imamura et al. 2010). Another study showed that the transgenic *Arabidopsis* encoded thanatin was resistant to the fungal and bacterial pathogens (Wu et al. 2013).

Development of transgenic plants with the ability of AMPs production has been proposed as a method to protect plants against pathogens. Despite the benefits of transgenic plants, these plants have some limitations and disadvantages that should not be ignored (Wolfenbarger and Phifer 2000; Andow and Zwahlen 2006). Therefore, due to the lack of permanent access to Amps sources and the associated challenges with transgenic plants, other production platforms resulting recombinant peptide were suggested. One of the most famous platforms for production of recombinant peptides is human embryonic kidney cells (HEK293) which are widely used as a powerful tool for expressing recombinant proteins (Thomas and Smart 2005; Hu et al. 2018).

In this study, HEK293 transfected in our pervious study (Tanhaeian et al. 2018) was used for the synthesis and release of recombinant thanatin into medium. In order to demonstrate the effectiveness of recombinant thanatin against fungal plant pathogens, it is necessary to compare the efficacy of this recombinant peptide with other materials having antifungal activity. For this reason, we have compared the efficacy of two plant extracts and a chemical mixture along with recombinant thanatin against five fungal plant pathogens. The results indicated that, all treatments have shown antifungal activity against tested fungi. Amongst all treatments, thanatin showed a good antifungal activity even by its application at µg level under in vitro and in vivo condition.

## Materials and methods

### Preparation of recombinant thanatin

Thanatin gene cloning processes was performed according to the same protocol which we used in our recent publication (Tanhaeian et al. 2018). Briefly, the plasmid vector harboring synthetic thanatin encoding sequence (shown in a Additional file 1) and pcDNA™3.1(+) vector (Thermo Fisher Scientific, USA) were digested using *Bam*HI restriction enzyme (Thermo Fisher Scientific, USA). Digested products were purified by gel extraction kit (Thermo Fisher Scientific, USA), and treated by *Xba*I restriction enzyme (Thermo Fisher Scientific, USA) for 5 h; retrieved from the gel and used for ligation by fast ligation kit (Thermo Fisher Scientific, USA). 10 μl of ligation product was then transformed into DH5α competent cells (Invitrogen, USA). The transformed colonies containing the recombinant plasmid (pcDNA™3.1(+)-thanatin) were cultured and subjected for plasmid extraction using a plasmid extraction kit (Thermo Fisher Scientific, USA). Transformation process was confirmed by restriction mapping and sequencing.

The adherent Human embryonic kidney 293 (HEK293) cell line was kindly provided by Department of Animal science, Ferdowsi University of Mashhad, maintained at 37 °C, 5% CO_2_ and 5% humidity in Dulbecco’s modified Eagle’s medium (DMEM/F12, Sigma Aldrich, USA) supplemented with 10% fetal bovine serum (FBS) (Fetal FCScalf serum from Gibco, USA) and 1% of the penicillin–streptomycin (Invitrogen, USA) as antibiotics. For transfection, the cells were cultured in 35 mm petri dishes and allowed to grow until making 50–80% confluent culture. Calcium phosphate co-precipitation procedure was performed in DMEM/F12 medium supplemented with 10% FBS and 1% of the penicillin–streptomycin. Recombinant vector and pcDNA™3.1(+) (without insertion, as a negative control) were transfected in separate dishes and then were selected using the medium containing 400 mg/mL Geneticin (Sigma-Aldrich, Germany) for 2 weeks. After antibiotic therapy, the transfected cells containing secretive thanatin were collected from the cultivated medium and stored at 5 °C.

### SDS-PAGE analysis

**Fig. 1:**
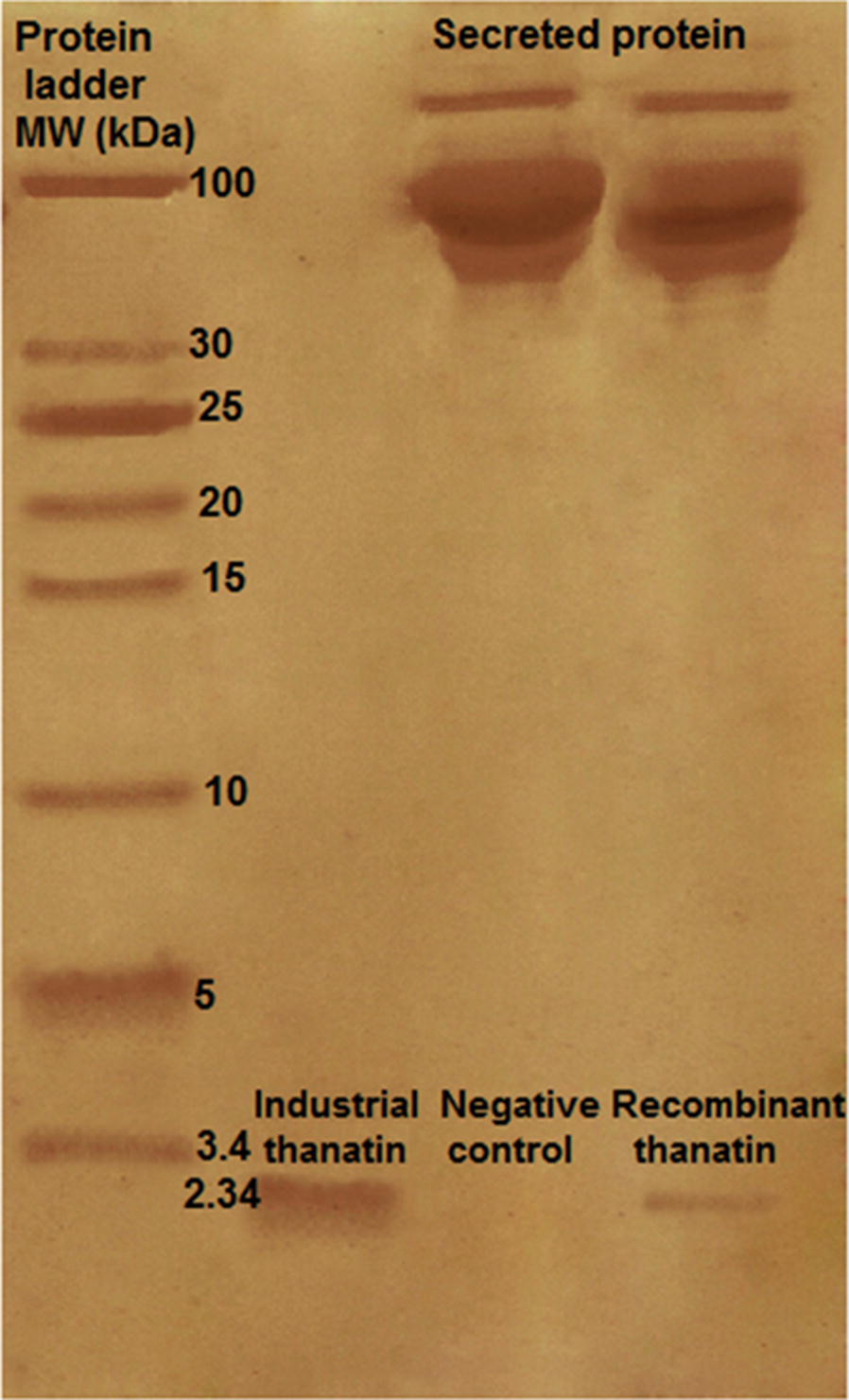
SDS-PAGE analysis of HEK293 secreted protein in medium alongside with a low molecular protein ladder, industrial thanatin and negative control. The peptide band with the size of 2.34 kDa represented thanatin

30 µL from the supernatant upon transfected cells were run on SDS-PAGE in Tris/glycine/SDS buffer using 15% polyacrylamide gels alongside to a low molecular protein ladder, industrial thanatin and negative control sample (Fig. 1). Thanatin was separated on SDS-PAGE and visualized by silver nitrate according to the staining protocol suggested by manufacturer. The quantification of peptide band was carried out by NIH Image software (ImageJ 1.34s; http://rsb.info.nih.gov/ij).

### Preparation of aqueous extracts from Turmeric and Persian lilac

The plants were collected and washed for the first time with tap water. The second wash was conducted by sterile distilled water and then dried under shade in airy condition away from direct light. Dried plants were converted to the powder using a milling machine.

For each 100 mL of water, 10 g of turmeric/Persian lilac powder were added to the sterile distilled water and placed on a shaker at 100 rpm for 24 h. Then, the mixture of water and plant powder were clean up with a filter paper on a Buckner funnel using a vacuum pump. In order to change the plant extract to the powder, the obtained liquid was placed in the oven at 45 °C for 24 h. After water evaporation, the remaining powder were collected and used to prepare a solution with 100 mg/mL concentration. The solutions were kept at 4 °C until use (Azimi et al. 2006; Mariita et al. 2011; Alo et al. 2012).

### Chemical fungicide mixture

We used a chemical mixture including Mancozeb (Dithane M-45^®^) 64% W/W as a non-systemic fungicide with protective action on contact and Metalaxyl (Ridomil^®^) 8% W/W with systemic function and inert ingredient 28% W/W, in order to compare its antifungal activity versus two plant extracts and thanatin. The fungicides were purchase from Al-Mahmood Company (Manama, Bahrain).

### Fungal isolates

Fungal isolates were provided by the fungal collection at department of plant protection, Ferdwosi University of Mashhad as listed in Table 1.

**Table 1:** Phytopathogenic fungi used in antifungal activity test

Name	Strain number	Isolated host	Disease
*Geotrichum candidum*	IBRC-M 30010^a^	Potato	Potato rot in storage
*Botrytis cinerea*	IBRC-M 30162^a^	Banana	Grey mold rot
*Rhizoctonia solani*	IRAN 2957C^b^	Potato	Stem canker
*Alternaria tenuissima*	Local isolate	Tomato	Early blight
*Gibberella fujikuroi*	NBRC 9976^c^	Rice	Bakanae disease

^a^Iranian Biological Resources Center

^b^Iranian Fungal Culture Collection

^c^NITE Biological Resource Center

### Antifungal activity test

The plant extracts were mixed with medium in order to evaluate their antifungal activity. After preliminary estimation, the following concentrations 0, 16.66 and 25 mg/mL were prepared. Sterile distilled water was used as a solvent to dilute the stock solutions and as a negative control in treatments as well. PDA (Potato Dextrose Agar, Merck, Germany) was used as a growth medium for fungal cultivation. The medium was prepared, autoclaved and let to be cool down until 45 °C at room temperature. The plant extracts were added to the petri dishes with 6 cm diameter and mixed to prepare a uniform solution for each plant extract concentrations. The fungal disks with 6 mm diameter were taken from the 1 week fungal culture using a sterile cork borer and placed in the middle of petri dishes. The petri dishes were incubated at 28 °C and the growth rate of fungi were measured every 24 h until the fungal mycelia were completely occupied the surface of PDA in control plates. Three replications for each concentration were considered for all fungal isolates (Hadian et al. 2006). The same procedures were performed in the plates amended with thanatin and chemical mixture with different concentrations. According to preliminary tests, we decided to use the following concentrations of thanatin; 0, 0.264 and 0.400 µg/mL and the concentration of chemical mixture were; 0, 0.001, 0.01 and 0.1 mg/mL. Thus, the required concentrations of thanatin and chemical fungicide were added to the medium. Subsequently, the rate of mycelial growth inhibition was calculated according to the following equation (Moslem and El-Kholie 2009):

$${\text{MGI }} = \, {{\left( {{\text{DC}} - {\text{DT}}} \right)} \mathord{\left/ {\vphantom {{\left( {{\text{DC}} - {\text{DT}}} \right)} {{\text{DC }} \times { 1}00}}} \right. \kern-0pt} {{\text{DC }} \times { 1}00}}$$

where, MGI: mycelial growth inhibition rate (%), DC: diameter of the control samples, DT: diameter of the test samples.

### Determination of MIC and MFC

The broth micro-dilution method (Irkin and Korukluoglu 2007; Plodpai et al. 2013) with some modifications was used to determine minimum inhibitory concentration (MIC) and minimum fungicidal concentration (MFC) of different plant extracts, thanatin and chemical mixture. Accordingly, a serial dilution was prepared from the stock solution of different treatments. Sterile distilled water was used as a solvent to dilute the stock solutions. PDB (Potato Dextrose Broth, Merck, Germany) was prepared, autoclaved and used as a basic medium for MIC/MFC evaluation. Then, the fungal disks were prepared using a cork borer from the marginal part of 7 days old fungal colonies and placed into the sterile empty test tubes containing 5 mL of PDB. One millilitre of different plant extracts, thanatin and chemical mixture at different concentrations were taken and added into the test tubes and mixed. The tubes were incubated at 25 °C for 5 days. Positive control tubes containing only broth media and fungal disk as well as negative control tubes containing broth media accompanied by 1 mL sterile distilled water was prepared and incubated at the same conditions. The MIC was defined as the lowest concentration of treatments that inhibited the visible growth of the fungi. In order to determine the MFC, the fungal disks were removed from the test tubes and sub-cultured on Petri dishes containing PDA medium and incubated at 25 °C for 5 days to determine if the inhibition was reversible. The MFC was the lowest concentration that did not permit growth on the plates.

### In vivo antifungal activity test

Tomato seedlings were used as host plants in order to investigate the potential efficacy of thanatin compared to the other material including plant extracts and chemical mixture. The plant pathogenic fungus *Alternaria tenuissima* causing early blight in tomato was selected as a test fungus.

The antifungal activities of different treatments were determined by a whole plant method in the greenhouse, as previously described by Bajpai and Kang 2010; Nashva and Abo-ElyouSr 2012). Briefly, the 7 week old tomato plants possessing an average of 3–5 leaves were kept in the green house with 14 h photoperiod and 60% humidity. The average temperature during day and night were 27 °C and 19 °C, respectively. The initial concentration of the test solutions were 1.6 µg/mL for thanatin, 100 mg/mL for both plant extracts and 2 mg/mL for chemical mixture. To prepare the test solutions, the stock solutions was diluted in 5 mL dimethylsulfoxide (DMSO) followed by dilution with the water containing Tween-20 (250 µg/mL). 30 mL from each solutions with different concentrations which were 1.6 and 0.8 µg/mL for thanatin, 25 and 50 mg/mL for both plant extracts and 1.5 and 2 mg/mL for chemical mixture, were individually sprayed as foliar application on tomato plants and repeated 15 days later. Two days after the second spraying, tomato plants were inoculated with 20 mL of *A*. *tenuissima* spore suspension containing 5 × 10^6^ spore/mL. After inoculation, plants were kept in a climate chamber at a daily temperature of 28 °C and 85% relative humidity. Disease development was recorded 15 days after inoculation. The severity of early blight was recorded for each treatment and scored from 0 to 9 according to the following rating system (0 = healthy plant, 1 = 1–5%, 3 = 6–10%, 5 = 11–25%, 7 = 26–50% and 9 ≥ 51% of the leaf area infected) suggested by Latha et al. (2009). In vivo experiments were repeated twice.

### Data analysis

All the analyses were performed in triplicate and data-sets were subjected to analysis of variance (ANOVA) and the Duncan’s multiple range test using SPSS 24 software. In all cases, a P value of ≤ 0.05 was considered significant. The diagrams were drawn using Microsoft Office Excel 2013.

## Results

### Transfected cell culture, thanatin expression and SDS-PAGE analysis

Cultivation of transfected HEK293 cells and expression of recombinant thanatin were successfully accomplished. A 2.34 kDa band corresponding to the size of thanatin and industrial thanatin was observed on the SDS-PAGE, confirming that the peptide was properly expressed in the host cells and secreted into medium (Fig. 1) as documented by Fehlbaum et al. (1996) and Koch et al. (2012). The pcDNA3.1+ vector without thanatin encoding sequence was transfected into HEK 293 cell as a negative control and the total secretion protein of this cell was run alongside the transfected sample carrying thanatin coding sequence. As observed there was no band with such a size in the control sample.

### Antifungal activity test

**Fig. 2:**
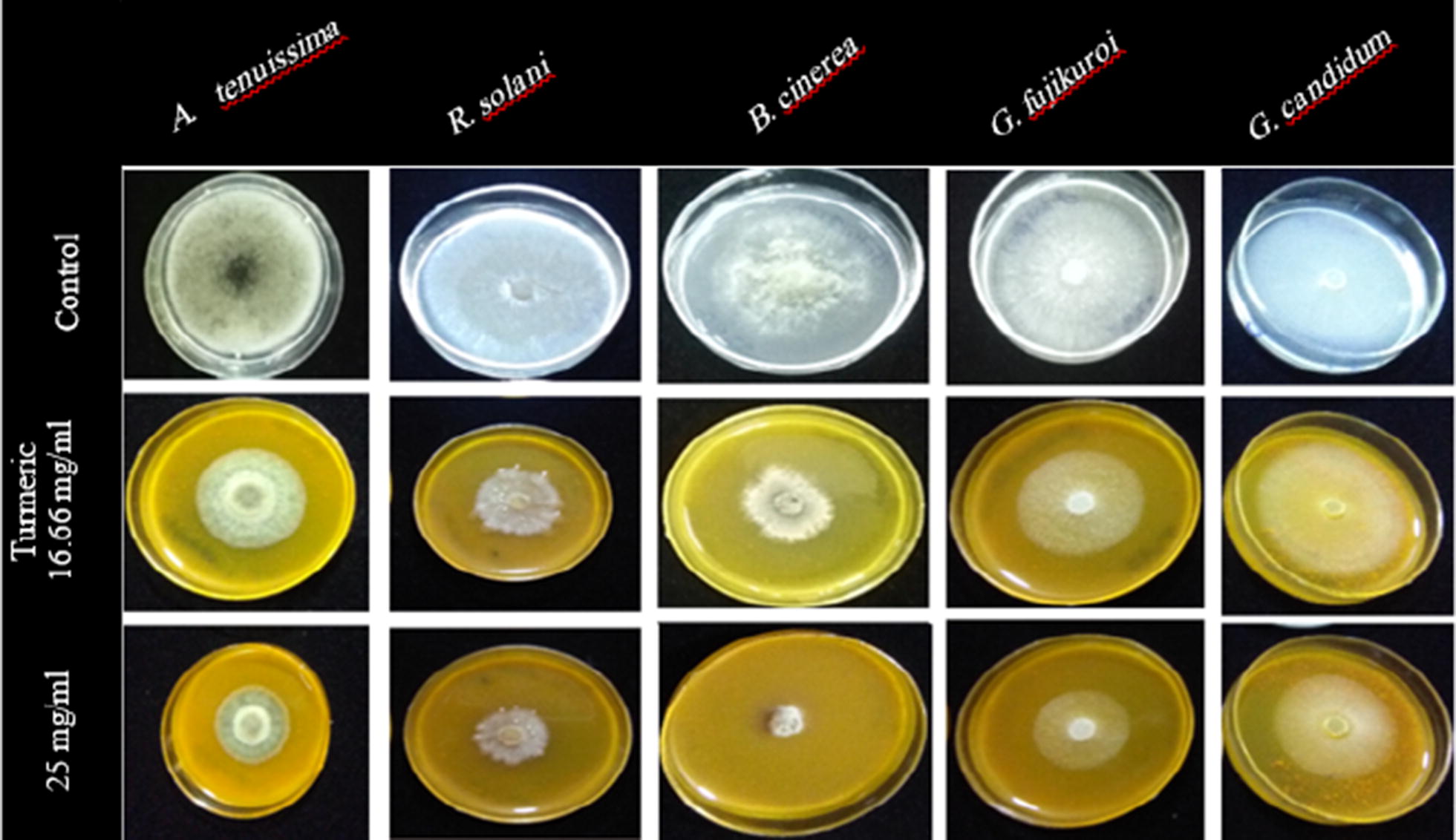
Antifungal effects of turmeric extract against different plant pathogenic fungi under in vitro condition

**Fig. 3:**
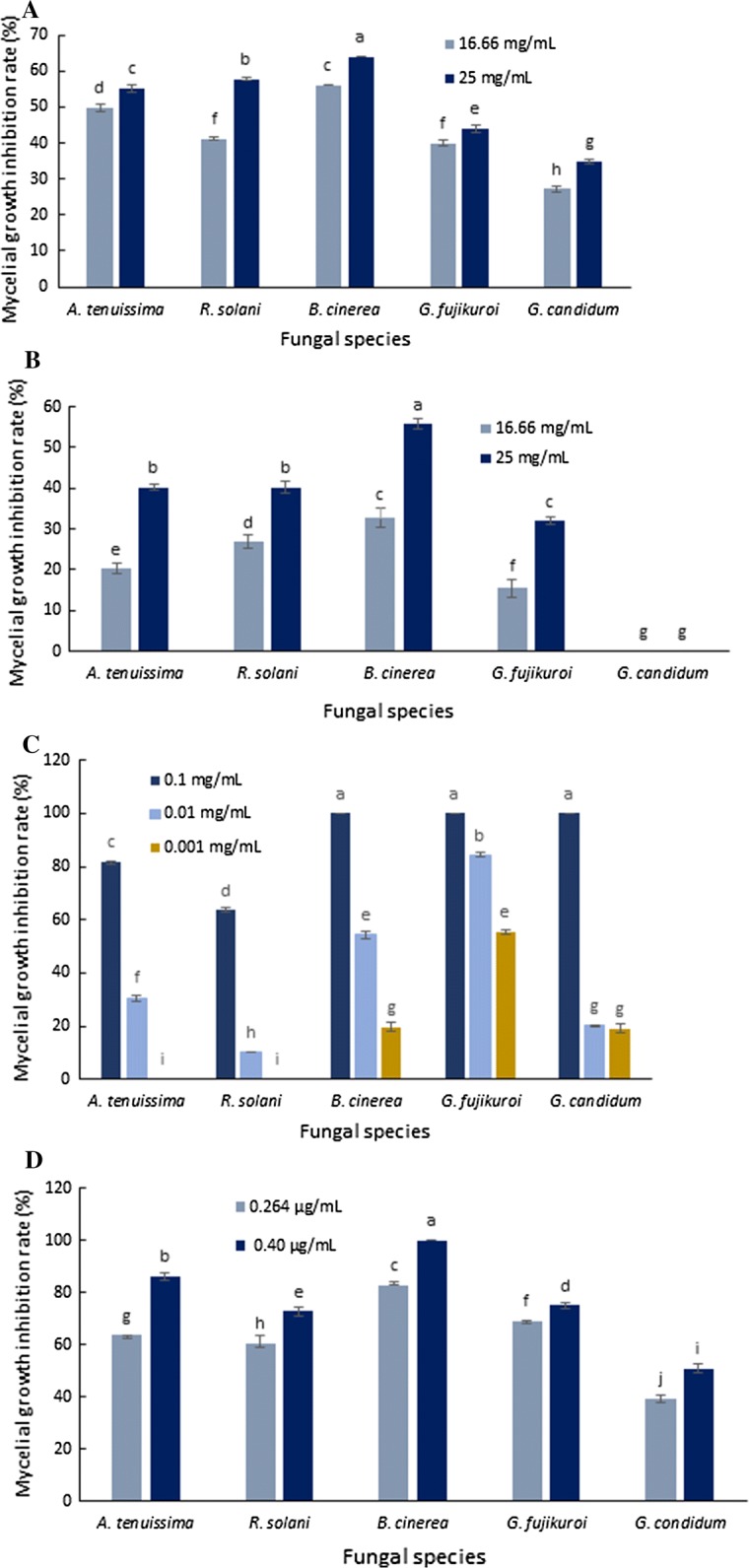
Mycelial growth inhibition of different fungal plant pathogen pretreated with turmeric extract (**A**), Persian lilac extract (**B**), chemical mixture contained Mancozeb + Metalaxyl (**C**) and recombinant thanatin (**D**)

**Fig. 4:**
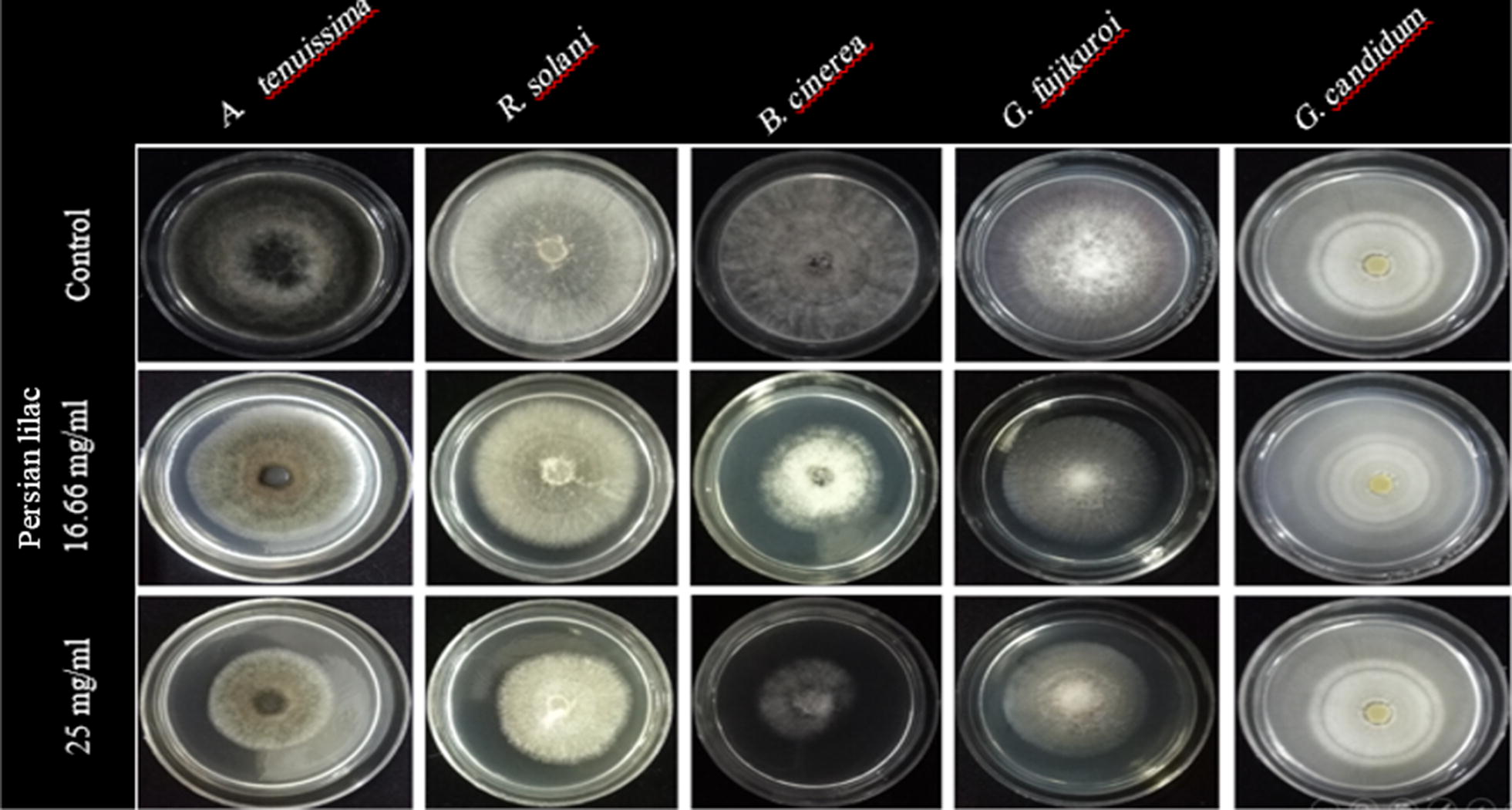
Antifungal effects of *Persian lilac* extract against different plant pathogenic fungi under in vitro condition

**Fig. 5:**
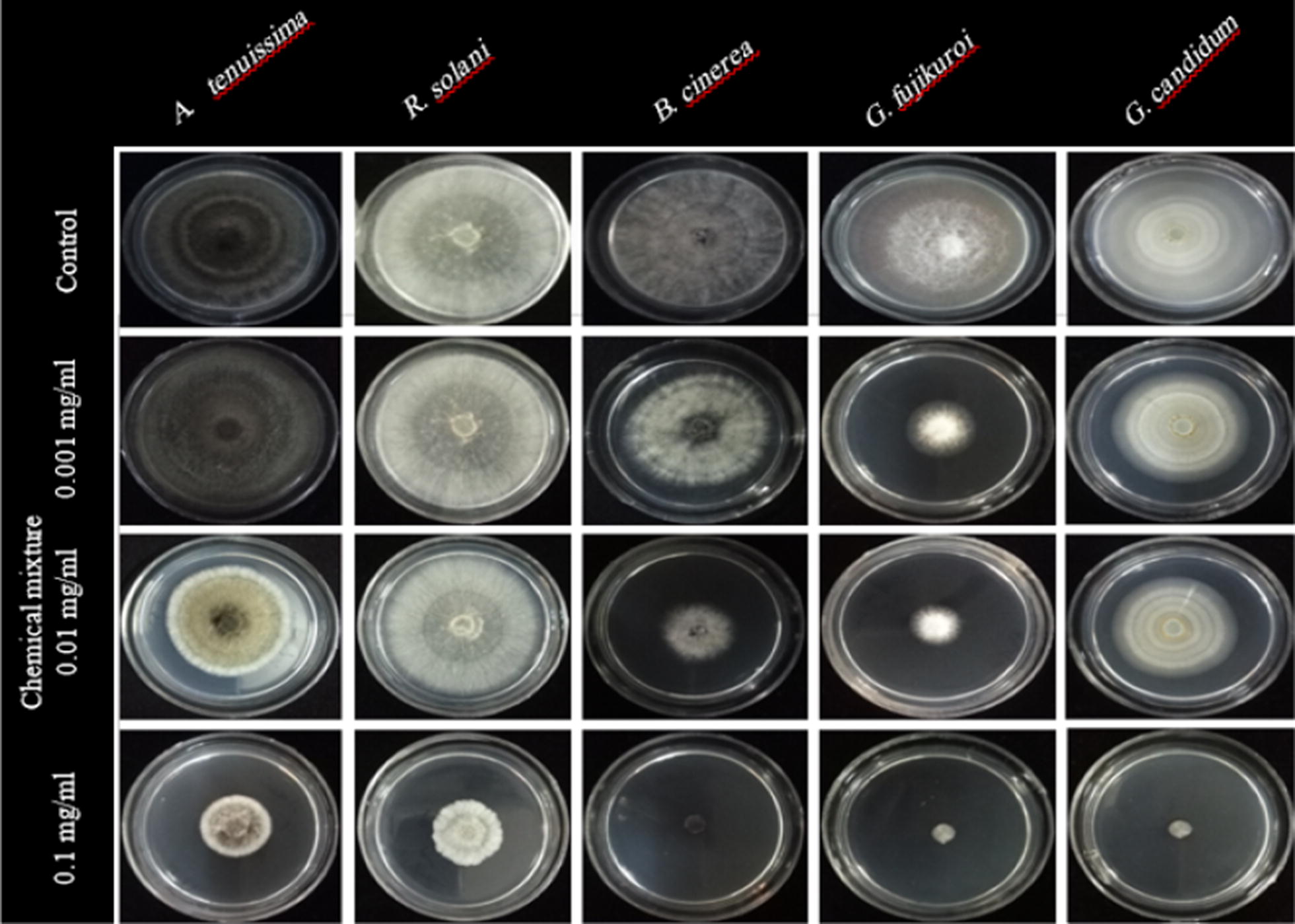
Antifungal effects of chemical mixture (Mancozeb + Metalaxyl) used against different plant pathogenic fungi under in vitro condition

**Fig. 6:**
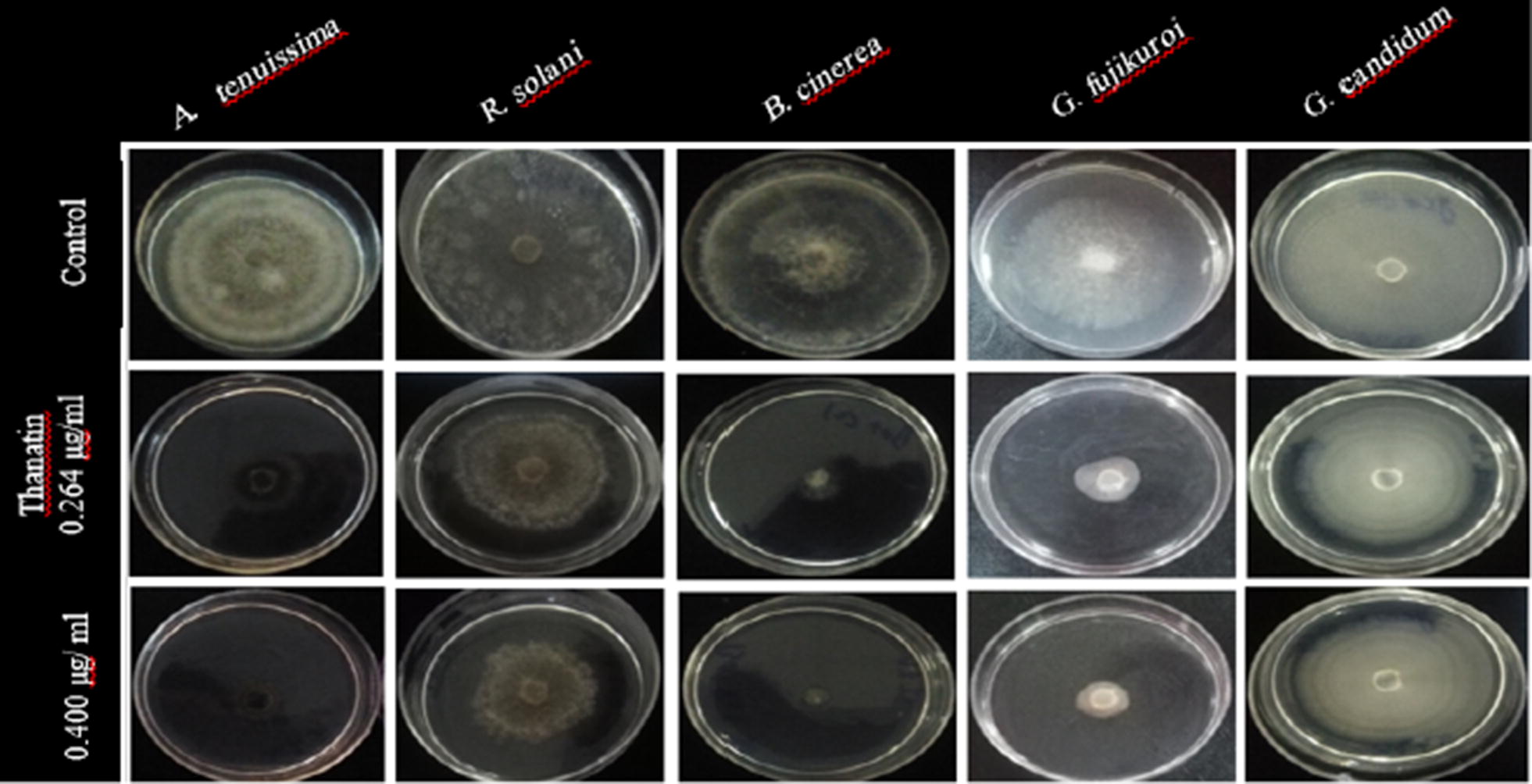
Antifungal effects of recombinant thanatin against different plant pathogenic fungi under in vitro condition

The results of antifungal effects and mycelial growth inhibition for turmeric, Persian lilac, chemical mixture and recombinant thanatin against all tested fungi have been presented in Figs. 2, 3, 4, 5 and 6, respectively. As shown turmeric has inhibited the growth rate of all tested fungi compared to the control samples in both applied concentrations (Fig. 2). The results of the Duncan test showed that, there is a significant difference between inhibition rates of *B. cinerea* compared to the other species. Basically, a higher rate of inhibition was observed in the higher application of turmeric extract in tested fungi. Turmeric at the concentration of 25 mg/mL had the highest amount of inhibition rate against *B. cinerea*. The lowest rate of inhibition was observed in the fungus *G. candidum* at 16.66 mg/mL concentration (Fig. 3A).

As has been presented, Persian lilac was inhibited the mycelial growth of all tested fungi excluding *G. candidum*, compared to the control samples in the applied concentrations (Fig. 4). In overall, the results of Duncan test showed that, higher rate of inhibition was observed in the higher concentration of Persian lilac extract in all tested fungi. The concentration of 25 mg/mL had shown the highest amount of inhibition rate against *B. cinerea*. There is a significant difference between the inhibition rates of *B. cinerea* compared to the other species. At 25 mg/mL concentration of Persian lilac, the fungi *A. tenuissima* and *R. solani* had shown no significant differences. The fungi *R. solani* and *G. fujikuroi* at 16.66 mg/mL concentration did not show any significant difference (Fig. 3B).

Regarding to the plant extracts, a simple comparison between Fig. 3A, B is showing that, turmeric has been found to be more effective than Persian lilac against tested fungi in similar concentrations.

As expected, chemical mixture was inhibited the mycelial growth of all tested fungi compared to the control samples in applied concentrations (Fig. 5). The results of Duncan test on chemical mixture indicated that, all treatments were significantly different in applied concentration. Obviously, by increasing of fungicide concentration, the rate of fungal inhibition will also be increased in tested fungi. The antifungal activity of chemical mixture at the concentration of 0.1 mg/mL in the fungal species; *B. cinerea*, *G. fujikuroi* and *G. candidum* were not significantly different. There are no significant difference between *B. cinerea* and *G. candidum* in 0.001 mg/mL concentration (Fig. 3C).

The results obtained from thanatin antifungal activity test showed that, similar to plant extracts and chemical mixture, by increasing of thanatin concentration, the rate of fungal inhibition will also be increased (Fig. 6). Interestingly, the applied concentrations of thanatin are not comparable with other three treatments. The highest and lowest inhibition rates were observed in the fungi *B. cinerea* and *G. candidum,* respectively (Fig. 3D).

### MIC and MFC results

The minimum inhibitory concentration and minimum fungicidal concentration of different treatments were determined for the five plant pathogenic fungi (Table 2). As presented, both plant extracts and thanatin have shown inhibitory effects against all tested fungi. Remarkably, the concentrations of MIC and MFC are showing that, the antifungal effects of thanatin are much stronger than that of the plant extracts and chemical mixture too. The results are clearly showing that, thanatin is more potent than the other treatments in controlling five fungal plant pathogens.

**Table 2:** Determination of MIC and MFC for different treatments tested against five fungal plant pathogen

Fungi	*A. tenuissima*		*R. solani*		*B. cinerea*		*G. fujikuroi*		*G. candidum*	
MIC/MFC (µg/mL)	MIC	MFC	MIC	MFC	MIC	MFC	MIC	MFC	MIC	MFC
Turmeric	6250	50,000	3250	25,000	3125	25,000	6250	50,000	12,500	100,000
Persian lilac	25,000	–	12,500	–	12,500	–	25,000	–	50,000	–
Chemical mixture	500	2000	500	2000	250	1000	250	1000	500	1500
Thanatin	0.6	1.2	0.6	1.2	0.14	0.6	0.3	0.6	1.2	2.4

### Antifungal evaluation under in vivo condition

**Fig. 7:**
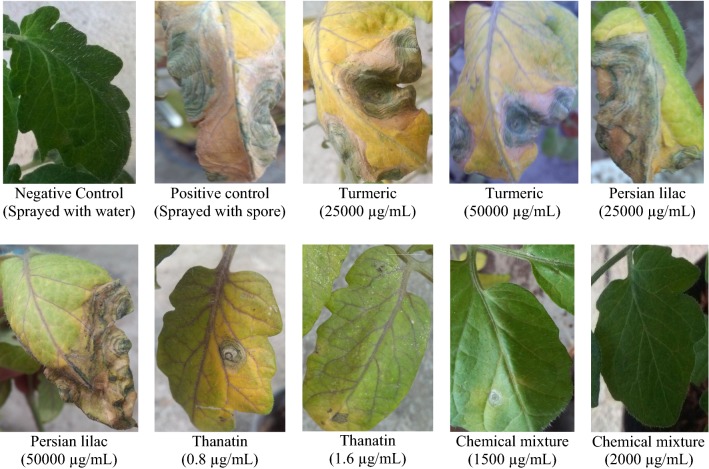
The response of different pretreated tomato plants 15 days after leaf inoculation by *A. tenuissima*

**Fig. 8:**
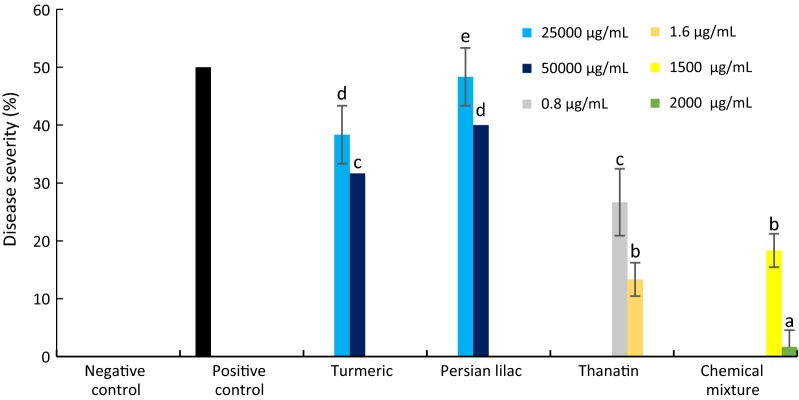
Severity of early blight disease evaluated on different pretreated tomato plants 15 days after inoculation by *A. tenuissima*

According to the results given in Fig. 7, the plant extract were shown a sensible antifungal activity under in vivo condition. Both plant extracts, significantly reduced the severity of fungal disease tomato early blight caused by *A. tenuissima* in applied concentrations (Fig. 8). The most effective treatments between plant extracts was turmeric at 50 mg/mL concentration. Among all treatments, thanatin presented a good controlling effects on tomato early blight under in vivo condition (Fig. 8). Notably, the inhibition effect of thanatin was observed by its application at µg level, while the inhibitory effects of plant extracts were observed by their application at mg level.

## Discussion

Human food security is threaten by different plant diseases and the quality and quantity of crops are reduced by several plant diseases which caused by diverse fungi, bacteria, nematodes, viruses and, etc. Amongst different plant pathogens, fungi are the most important organisms caused plant diseases so that, their control is unavoidable. The most commonly used method for inhibiting plant fungal pathogens is the application of chemical fungicides that are considered as environmental pollutants and also as potential threats to human health and environment (Damalas and Eleftherohorinos 2011; Andersson et al. 2014). Furthermore, plant pathogens are getting resistant to many of these chemicals fungicides (Hahn 2014). Consequently, the attention of many researchers has been drawn to the other alternatives and harmless materials with antifungal activity. For example, several studies have been accomplished on the application of essential oil and plant extracts to inhibit fungal plant pathogens (Sales et al. 2016).

In the present study, the antifungal activity of two plant extracts named turmeric and Persian lilac along with a chemical mixture and recombinant thanatin were evaluated against following fungal plant pathogens; *G. candidum*, *B. cinerea*, *R. solani*, *A. tenuissima* and *G. fujikuroi*. The obtained result confirmed the antifungal activity of all treatments against tested fungi. Between two plant extracts, turmeric showed a higher rate of mycelial inhibition against tested fungi than Persian lilac. Although, the antifungal activity of plant extracts was satisfactory, the evaluated MIC and MFC values for them were much higher than that of chemical mixture which means two plant extracts were basically not comparable with chemical mixture (Table 2). However, in terms of maintaining health and environmental aspects, they are much more preferable than chemical fungicides. As a result, the aqueous extracts of turmeric and Persian lilac can be considered as good alternatives to control fungal plant disease as previously documented by Damalas 2011 in case of turmeric.

Another suggestion is to utilize from the potential of antimicrobial peptides (Datta et al. 2015). In this regard, thanatin is one of the most appropriate antimicrobial peptides that we would like to offer as a promising compounds to control fungal plant diseases. The valuable features of thanatin, such as being non-allergic for human (Wu et al. 2011) made this peptide encoding sequence as a good candidate to be insert in plants for making transgenic resistance crops against fungal pathogens (Wu et al. 2010, 2013; Imamura et al. 2010, 2016). However, due to the limitations and disadvantages of transgenic plants (Lu 2008; Prakash et al. 2011), synthetizing of thanatin in the form of recombinant and secretion in HEK293 can be more efficient and manageable in the wide scale. It should also be mentioned that, the cost of production for this antimicrobial peptide is not comparable to chemical fungicides. Regarding to the great antifungal and antibacterial (Mamarabadi et al. unpublished data) activity of thanatin and its compatibility with human health and environmental safety compared to unpleasant effects of chemical fungicides, we could imagine a clear perspective for this peptide with the intention of its application in sustainable agriculture.

## Additional file

**Additional file 1.** Schematic representation of thanatin nucleotide and amino acid sequences.
