# Supplementary figures and images for: Antifungal activity of recombinant thanatin in comparison with two plant extracts and a chemical mixture to control fungal plant pathogens
Source: AMB Express. 2018 Nov 2;8:180. doi: 10.1186/s13568-018-0710-4 (PMC6214488; doi:10.1186/s13568-018-0710-4)

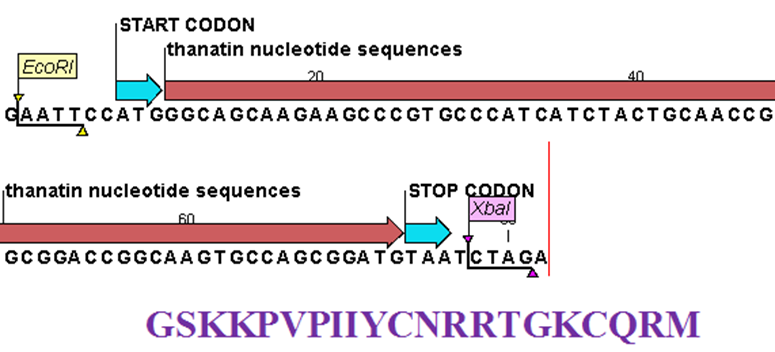


Schematic representation of thanatin nucleotide and amino acid sequences.

Supplement: Supplementary file 1 — Additional file 1. Schematic representation of thanatin nucleotide and amino acid sequences. [file 13568_2018_710_MOESM1_ESM.docx]
